# Adaptation of a Theory-Based Social Networking and Gamified App-Based Intervention to Improve Pre-Exposure Prophylaxis Adherence Among Young Men Who Have Sex With Men in Bangkok, Thailand: Qualitative Study

**DOI:** 10.2196/23852

**Published:** 2021-11-04

**Authors:** Wipaporn Natalie Songtaweesin, Sara LeGrand, Shashika Bandara, Caitlin Piccone, Prissana Wongharn, Juthamanee Moonwong, Thidarat Jupimai, Chutima Saisaengjan, Tuangtip Theerawit, Kathryn Muessig, Lisa Hightow-Weidman, Thanyawee Puthanakit, Nittaya Phanuphak, Arunrat Tangmunkongvorakul

**Affiliations:** 1 Faculty of Medicine Center of Excellence for Pediatric Infectious Diseases and Vaccines Chulalongkorn University Bangkok Thailand; 2 Center for Health Policy and Inequalities Research Duke Global Health Institute Duke University Durham, NC United States; 3 Department of Health Behavior Gillings School of Global Public Health University of North Carolina at Chapel Hill Chapel Hill, NC United States; 4 Behavior and Technology Lab Institute for Global Health and Infectious Diseases University of North Carolina at Chapel Hill Chapel Hill, NC United States; 5 Department of Pediatrics Faculty of Medicine Chulalongkorn University Bangkok Thailand; 6 Institute for HIV Research and Innovation Bangkok Thailand; 7 Research Institute for Health Sciences Chiang Mai University Chiang Mai Thailand

**Keywords:** mobile health, young men who have sex with men, pre-exposure prophylaxis, adherence, mobile phone

## Abstract

**Background:**

HIV disproportionately affects young Thai men who have sex with men (YMSM). Recent studies report a high incidence and prevalence of HIV among Thai YMSM. The Thai national guidelines have recommended pre-exposure prophylaxis (PrEP) since 2014 for key populations; free PrEP has been piloted since 2019. Smartphone-based mobile health (mHealth) interventions provide an optimal platform for innovative PrEP adherence interventions for Thai YMSM.

**Objective:**

This study aims to adapt the P3 (Prepared, Protected, emPowered) app, developed with YMSM and transwomen in the United States to improve PrEP adherence and persistence for YMSM in Thailand. The app aims to provide daily adherence support and addresses gaps in staff available for large-scale PrEP rollout needed to see population-level effects of HIV prevention.

**Methods:**

We conducted focus group discussions (FGDs) with YMSM and key informant interviews (KIIs) with PrEP care providers in Bangkok, Thailand, to investigate PrEP adherence facilitators and barriers, preferences for functions and features in mHealth apps among YMSM, and how to best adapt the P3 app to the Thai context. We conducted four FGDs with 4-8 participants per group and 15 KIIs.

**Results:**

For FGDs, 23 YMSM participated with a mean age of 20 years (range 18-21), 96% (22/23) enrolled in full-time education, and all owned smartphones. The mean age of KII participants was 40 (range 26-60) years; most were state health service providers, with the majority being counselors (6/15, 40%) and physicians (6/15, 40%). Overall, the facilitators and barriers for PrEP adherence identified were similar to those of MSM and YMSM globally including the United States. Key themes included general recommendations for improving mHealth apps in Thailand, such as presenting reliable information in an appealing format, minimizing privacy risks, and addressing connectivity challenges. Additional themes focused on P3 Thailand adaptations and were related to cultural and stylistic preferences, engagement strategies, and recommendations for new functions. To develop the adapted app, P3 Thailand, these findings were balanced with resource limitations resulting in the prioritization of minor modifications: changes in app esthetics (color scheme, iconography, and imagery) and changes in the presentation of information in two of the app’s features. FGDs identified similar PrEP adherence facilitators and barriers to those already addressed within the app.

**Conclusions:**

The core elements of the P3 app address major PrEP facilitators and barriers for Thai YMSM; however, changes to the app features, including stylistic presentation, were needed to appropriately customize the app to the Thai context. Given the similarities of facilitators and barriers for PrEP adherence globally, adapting existing PrEP mHealth solutions based on input from end users and key informants provides a promising approach. However, partnerships with local app designers and developers can improve the adaptation process and final product.

**Trial Registration:**

ClinicalTrials.gov NCT04413708; http://clinicaltrials.gov/ct2/show/NCT04413708

## Introduction

In Thailand, gay, bisexual, and other men who have sex with men (MSM) are disproportionately affected by HIV [1]. Studies conducted in Bangkok have identified a high HIV incidence and prevalence among MSM, with young MSM (YMSM) at a significantly greater risk of HIV infection than older MSM [2-4]. Examinations of data from YMSM aged 18-24 years enrolled in the Bangkok MSM Cohort Study (40.8%; n=712) between 2006 and 2014 found a baseline HIV prevalence of 21.2% and an overall HIV incidence of 7.4 per 100 person-years, a rate much higher than that for MSM of all ages (5.3 per 100 person-years) [3,4]. To address disparities in HIV infection among YMSM in Thailand, the implementation of novel behavioral and biomedical interventions that are culturally and developmentally appropriate, useful, and scalable are urgently needed [4,5].

Pre-exposure prophylaxis (PrEP), a combination drug containing tenofovir and emtricitabine taken once daily, is a safe and effective method for preventing transmission of HIV among MSM [6-8]. Owing to the high levels of efficacy found in PrEP trials, the Thai National Guidelines on HIV/AIDS Treatment and Prevention in 2014 were revised to recommend PrEP for key populations, including YMSM [9,10]. Since October 2019, a coverage scheme providing free PrEP for key populations, including YMSM, has been piloted in Thailand’s National Health Security Office, with a view for future scale up [11,12]. Although PrEP availability is expanding rapidly for Thai YMSM, PrEP effectiveness is highly correlated with adherence, and evidence suggests poorer adherence among YMSM, including those in Thailand [7,13-16]. For example, two studies evaluating the Princess PrEP program, the first key population-led PrEP initiative in Thailand, found lower levels of PrEP adherence among MSM participants aged <25 years compared with those aged >25 years [13,14].

Smartphones provide an optimal platform for the delivery of an innovative PrEP adherence intervention in addition to other chronic medical conditions in adolescents because they are an integral part of their lives, including Thai YMSM [14,17-21]. There is growing evidence to support the improvement in self-management related outcomes in adolescents using electronic health interventions, in addition to user feasibility, acceptability, and satisfaction of such interventions [20,22,23]. Data collected by the Thai Red Cross AIDS Research Center (TRCARC) from 2015 to 2017 on the use of social networking sites by MSM aged >18 years and at risk of HIV (n=465) showed that users were satisfied with web-based service delivery primarily delivered through smartphones [24]. In addition, app-based HIV prevention interventions have shown high rates of acceptance among MSM, including YMSM, in high-income countries and have potential use in low-middle income countries [25-29]. On the basis of high smartphone use and findings from other study teams in Thailand and globally, app interventions are likely to be acceptable, help to overcome barriers to engagement with in-person interventions such as inconvenience, transportation, and stigma; and address challenges associated with medication, including PrEP adherence and other HIV prevention behaviors among adolescents, including YMSM in Thailand [13,14,17,22-24,30].

Rather than building de novo smartphone HIV prevention apps, there is growing support for adapting and building on existing evidenced-based platforms that share common behavior change goals, theoretical underpinnings, and features to maximize the potential for intervention sustainability, affordability, and scalability [31-33]. As such, this study adapted the theory-based P3 (Prepared, Protected, emPowered) app designed by researchers at the University of North Carolina at Chapel Hill and Ayogo Health, Inc [33]. The P3 app aims to improve PrEP adherence, retention in PrEP clinical care, and PrEP persistence among YMSM and young transgender women who have sex with men in the United States. To achieve its aims, P3 uses social networking and game-based elements along with numerous evidenced-based features, including social cognitive theory, narrative communication, and principles of the Fogg behavioral model of persuasive technology to promote behavioral change through triggers, ability, and motivation [34]. The purpose of this study is to conduct formative research to guide adaptations to P3 that will optimize app relevance, user engagement, and utility of the adapted app for Thai YMSM, henceforth known as P3 Thailand (P3T). We hypothesize that stylistic and cultural preferences will be different from US YMSM preferences, particularly with app esthetics and preference for more visual content and less text.

## Methods

### Overview

To adapt the P3 app to the Thai context, we collected qualitative data through focus group discussions (FGDs) with YMSM and key informant interviews (KIIs) in Bangkok, Thailand, between January and March 2019. Participants in both the FGDs and KIIs (1) described facilitators and barriers to PrEP adherence among Thai YMSM, (2) discussed mobile health (mHealth) app features and functions preferred by YMSM; and (3) provided specific feedback about P3 and offered suggestions for adapting it to create P3T.

Institutional review board approval was granted for this study by the Faculty of Medicine, Chulalongkorn University, with a waiver for parental consent granted. This study was registered with ClinicalTrials.gov (NCT04413708).

All study participants completed a brief computer-assisted self-interviewing survey on patient demographics, technology use, and previous experience using or providing PrEP before their session. All FGDs and KIIs were facilitated by a study staff member using a semistructured interview guide. After exploring PrEP adherence facilitators and barriers and mHealth app preferences among Thai YMSM, the facilitator gave a presentation of key features of P3 and elicited feedback and suggested changes from study participants.

The presentation of P3 features included screenshots and descriptions translated into Thai by the facilitator or interviewer starting with screenshots of onboarding activities (eg, review of community guidelines, app terms and conditions, setting up an account, option to lock screen with a PIN, brief introductory screens describing key P3 features, and profile setup). Next, participants were shown a screenshot of the user dashboard, or home screen, with an explanation of each icon. Five key features of P3 (Textbox 1) were demonstrated using screenshots and explained in detail by the facilitator, including the social wall, daily quests, medication tracking and adherence support, knowledge center, and collections. An additional key feature, adherence counseling, was not presented via screenshots but was explained during the presentation of the user dashboard screenshot. A brief description of these key features is provided in Textbox 1. Detailed information on all P3 features has been described elsewhere [34].

**P3 features discussed in focus group discussions and key informant interviews to adapt for Prepared, Protected, emPowered Thailand.**

**Key Prepared, Protected, emPowered** (**P3**) **features discussed in detail in focus group discussions and key informant interviews:**

Social wall:

- Daily prompts (eg, “Share tips for being safe if you are having a lot of sex!”) provide a safe space for discussions on topics related to pre-exposure prophylaxis (PrEP) adherence and sexual health such as mental health and positive sexual health habits
- Peer sharing, support, and reinforcement are intended to foster behavior change and build a sense of community among users
- Users are sent push notifications when someone has commented on or *liked* a post

Daily quests:

- Routine tasks that help users change behaviors by building knowledge and skills and setting goals (eg, “Why did you decide to start taking PrEP? Consider your reasons for starting PrEP and write them down. Knowing your reasons for taking PrEP can help motivate you!”)

Medication tracking and adherence support:

- To promote medication-taking habits, users personalize adherence strategies based on the time or circumstances in which they plan to take their medication (eg, “I take PrEP when I brush my teeth”)
- The medication (med) reminder system provides discreet and focused reminders based on user-selected time and adherence strategies. Reminders are linked to a med check prompt that allows users to track adherence or nonadherence for the past 3 days; data entered are used to update the in-app calendar, which shows adherence history. Users can also access the med check prompts and adherence history feature from the home screen
- Adherence support: The study team can monitor users’ PrEP adherence and app use. Encouraging messages can be sent to the user to provide support and encouragement

Knowledge center:

- A multimedia library with information on PrEP use, frequently asked questions about PrEP, PrEP side effects, and sexual health–related topics. A progression bar shows completion of each section
- Users can read articles to build their knowledge, respond with one of three responses indicating the usefulness of the article, and answer questions that encourage personal reflections about the content (not visible to other app users)

Collections:

- A choose-your-own-adventure style story for the user that closely mirrors choices and circumstances they may face in their daily lives
- Playing through these storylines allows users to build problem-solving skills when faced with difficult choices
- The *collections* feature also acts as an incentive for users to engage with other features of the app to gain points to unlock stories

Adherence counseling:

- Personalized adherence counseling is provided via secure in-app messaging; addresses adherence challenges faced by young men who have sex with men
- Key features include reviewing sexual risk experiences of the participants, exploring PrEP adherence facilitators and barriers, identifying adherence needs and strategies to meet needs, discussing mental health and relationship issues, and developing an adherence action plan [35,36]

### Recruitment for FGDs

FGD participants were recruited from (1) HIV prevention clinics at the Center for Excellence for Pediatric Infectious Diseases at the Chulalongkorn University, a major teaching hospital in Bangkok; (2) TRCARC, Thailand’s largest HIV voluntary counseling and testing (VCT) center, which serves over 2000 adolescents per year; and (3) Two community-based organization branches of TRCARC, Service Workers IN Group and Rainbow Sky Association Thailand. Potential participants were approached at HIV VCT visits to assess their interest in study participation. In addition, study advertisements were posted in clinics, social media, and via the TRCARC website. The inclusion criteria were as follows: (1) age 16-24 years, (2) male sex at birth, (3) self-identification as MSM, (4) ability to speak and read Thai, (5) familiarity with smartphones, and (6) current or previous PrEP use. Those unable to provide consent due to substance use or psychological conditions were excluded.

FGDs lasting 60-120 minutes were conducted in private conference rooms either at offices in clinics or reserved private conference rooms in cafes in groups of 4-8. All participants were identified using a pseudonym to protect their confidentiality. At the conclusion of the FGDs, participants were provided THB 500 (US $16) to compensate them for their time.

### Recruitment for KIIs

Key informants who had collaborated with the Center for Excellence for Pediatric Infectious Diseases or participated in academic conferences on adolescent health in Bangkok were recruited for KIIs via in-person contact, phone calls, text messages, and email invitations. To participate in a KII, individuals were required to be a current PrEP provider for YMSM in Bangkok. KIIs, which lasted between 40 and 60 minutes were conducted face-to-face at PrEP clinics. The KII participants were not compensated.

### Data Analysis

Pre-FGD and KII survey data were summarized using Adobe Acrobat. FGDs and KIIs were audio-recorded and transcribed in Thai. A directed content analysis approach, defined as categorization derived directly from text data and transcribed interviews, was used [37]. An initial codebook was developed collaboratively between the US and Thai study teams based on semistructured interview guides that reflected the study research themes and subthemes. An iterative approach was used when new codes arose, as interviews were conducted. New codes emerging were added, and any transcribed code that had already been coded was recoded. Two members of the Thai study team coded the FGD and KII transcripts using NVivo (version 12.5.0) [38]; statistical interrater reliability for coding was not calculated. Instead, divergence among coders was discussed among the analysis groups to reach consensus, resolved by the Thai site principal investigator. Data were then deductively analyzed using thematic analysis by the same Thai study team members to identify key themes and subthemes relevant to the adaptation of P3 for YMSM in Thailand. Quotes illustrative of key themes were identified and translated into English by bilingual Thai English speakers.

## Results

### Participant Characteristics

#### Characteristics of FGDs

A total of 23 YMSM participated in four FGDs. Participants were aged 18-21 years, with a mean age of 20 years. The majority were enrolled in full-time education 96% (22/23), with 70% (16/23) enrolled in university. All participants owned a smartphone and used it to access the internet; 74% (17/23) were on self-paid monthly internet plans. Over half of the participants (13/23, 57%) had phones that used an iOS (Apple iPhone operating system) and 35% (8/23) used an Android operating system. Most (22/23, 96%) were current PrEP users and 4% (1/23) were previous PrEP users.

#### Characteristics of KIIs

A total of 15 KIIs were conducted; participants were aged 26-60 years with a mean age of 40 years. Most were counselors (6/15, 40%) and physicians (6/15, 40%); the remaining 20% included 2 nurses and 1 social worker. All patients were employed at publicly accessible HIV VCT clinics. The majority of key informant interviewees had completed university level education (11/15, 73%), and 60% (9/15) had been providing PrEP to adolescents for 3-4 years.

### Overview of FGD and KII Findings

#### General Overview

Key findings from the FGDs and KIIs were grouped into two main categories. The first focuses on the facilitators and barriers to PrEP adherence among YMSM in Bangkok. The second focuses on general and specific recommendations for ensuring P3T app relevance, user engagement, and utility for Thai YMSM. We present these themes and subthemes along with English translations of illustrative excerpted participant quotes.

#### PrEP Adherence Facilitators and Barriers

##### FGD Findings: Adherence Facilitators

Key subthemes that emerged regarding facilitators of PrEP adherence discussed by participants included risk perception, PrEP cost, and social support.

##### Risk Perception

FGD participants who felt they were at risk of HIV infection were more motivated to take PrEP:

I had a pretty risky history before. I use my history to determine my risk so now take it (regularly). I also take it because there were people that recommended it to me.

FGD1, Participant 3

##### PrEP Cost

PrEP being available free of charge was also important in supporting PrEP adherence:

It really helps with us being students [that PrEP is free] as it helps us save...if we had to pay for it, I would have to think a lot more whether or not to take it.

FGD4, Participant 6

##### Social Support

Social support from staff was another subtheme that FGD participants gave importance to in their willingness to adhere to PrEP:

For me what’s important is staff that are constantly available for counseling. If we had providers that just gave us our meds and that was the end of it—no questions about how we were, I wouldn’t want to come for PrEP services.

FGD4, Participant 3

#### KII Findings: Adherence Facilitators

The key facilitators of PrEP adherence discussed by key informants were similar and included social support as illustrated in the quote below.

For some couples who both take PrEP and are open to each other about this and have an open relationship, taking PrEP in front of each other does not need to be hidden for fear of being accused of being unfaithful.

KII, Participant 14

#### FGD Findings: Adherence Barriers

Subthemes that arose on the issue of major barriers for YMSM in PrEP adherence included stigma, logistical issues, and PrEP side effects.

##### Stigma

YMSM felt that taking PrEP was associated with stigma, risky sexual behavior, being gay, and also being seen to be infected with HIV:

People will feel that [PrEP] clinics are for gay men with risky behaviors. It’s well known in Bangkok that a lot of gay men are infected with HIV...people look at gay men negatively as it is...that we’re promiscuous.

FGD4, Participant 5

##### Logistical Issues

Logistical issues, including being busy with other activities, also featured prominently in reasons YMSM felt it was difficult to always adhere to PrEP:

We have a lot of classes and have to study for exams, there isn’t always time to come for appointments.

FGD2, Participant 3

##### PrEP Side Effects

PrEP side effects were another issue adolescents felt made PrEP adherence challenging, particularly when it interfered with other activities in their daily lives:

I had a lot of side effects with PrEP, and I have to study everyday—it makes it impossible to study.

FGD3, Participant 1

#### KII Findings: Adherence Barriers

Findings of major barriers from the point of view of KIIs were very similar to those of YMSM as already discussed above, including logistical issues, risk perception, and peer influence.

KIIs experienced difficulties arranging logistics to come for appointments and low HIV risk perception, in addition to a number who initiate PrEP because of peer influence and discontinue it because of lack of intrinsic motivation or sense of self-risk perception for HIV:

Many kids who don’t turn up to appointments will say its inconvenient for them to come in, they don’t have time to come, or they are no longer at risk. Some say they want to come but borrow PrEP from friends...confess they don’t have time for appointments but initially came because they saw their friends taking PrEP and wanted to join in, we see this a lot.

KII, Participant 2

#### Adaptations Made Based on PrEP Adherence Facilitators and Barriers

As the facilitators and barriers found in this study were essentially the same as those seen in the US P3 app, no changes were made to the app regarding this [34].

#### General and Specific Recommendations for Ensuring P3T App Relevance, User Engagement, and Utility for Thai YMSM

Six main themes emerged under this category: (1) information provided in mHealth apps, (2) stylistic preferences, (3) engagement strategies, (4) privacy risks, (5) connectivity challenges, and (6) recommendations for new functions. The first 3 themes focused on general recommendations for mHealth apps in Thailand based on discussions before the review of P3 features. The next 3 themes specifically focused on feedback and suggested adaptations to make the app more relevant to Thai users based on the review of P3 features.

#### Information Provided in the App

Three main subthemes related to app information emerged: (1) access to reliable information, (2) presentation of information, and (3) space for peer influence and information sharing.

#### Access to Reliable Information

In FGD findings, Several FGD participants discussed the ways in which Thai societal norms can be restricted to YMSM seeking information related to sexual health. However, they felt that access to such information was vital to self-care:

I think an app will help us feel less afraid to ask...sometimes society leads [us] to believe certain things are not okay [to ask] but it is actually beneficial. We want more freedom to be able to get hold of simple information to be able to look after ourselves. A lot of us worry looking after ourselves is too difficult and complicated.

FGD1, Participant 5

Despite the vast amount of information about PrEP available in Thai on the internet and social media sites, FGD participants were concerned that many sources lack reliability:

...it’s better to have accurate information, for example, answers to questions from doctors, [but] it’s so difficult to find official medical websites. Most of what I find is opinion sharing of the general public [on social media]...this is a problem.

FGD3, Participant 2

#### Presentation of Information

##### FGD Findings

Most FGD participants commented on the importance of the presentation of information in increasing app interest and engagement. They indicated a strong preference for information that was presented concisely and was visually oriented:

I think [information] should be [presented as] small nuggets of information, and when you click on it, it takes you to...an infographic, like a poster with concise information.

FGD1, Participant 1

Most teenagers will stop to read if there are graphics, but they will not read it if there is just text.

FGD2, Participant 6

In addition, participants highly recommended including video content and links to useful content outside the app.

When I open anything in Facebook, I see some interesting clips on pages for gay people—they will provide information about this stuff. If they have a collection of links of places to chill out, or links to You Tube videos, it just makes it more interesting.

FGD1, Participant 2

##### KII Findings

Another presentation style suggested was the use of social media influencers who use diverse methods to communicate information that is more appealing to YMSM:

I think having influencers on there with different messages. I’ve seen pictures on Instagram and Facebook and I think going there and being able to see their favorite movie star or whoever influences the community.

KII, Participant 11

#### Peer Influence and Information Sharing

##### FGD Findings

Participants noted the strong influence peers have on their decision-making; therefore, peers were viewed as key facilitators for app uptake and sustained engagement:

Thai teenagers listen much more to the opinion of their friends than making decisions on their own.

FGD3, Participant 4

I think if teens can see what other people think [is] worth reading, it would make me like, oh that looks like something I want to read, why are there so many people reading this?...like getting recommendations from others about it.

FGD 1, Participant 4

Sometimes teenagers may just want to talk to their friends or want information from them,-for example, who is taking PrEP and how they found it. People with previous experience will flood in to answer these queries, like on how they manage side effects. They tend to want experiential information from real-life users, which they find more convincing than information from doctors.

KII, Participant 2

##### KII Findings

Key informants also discussed the powerful nature of platforms for youth to connect with one another. They have the potential to provide a safe space for youth to discuss and share information about health-related topics that may be difficult to talk about in other settings while also creating opportunities to discuss similar life experiences with others:

I once had a closed online group for teenagers—this group were constantly talking. They would talk about health, then they would talk about something else, then back to health and medicines...an app could be potentially great for teens supporting each other towards a shared goal.

KII, Participant 1

#### Stylistic Preferences

##### FGD Findings

The presentation of key P3 features in the FGDs led to rich discussions about the *look and feel* of the app and its features. The app screenshots presented to study participants showed the P3 superhero theme, which included sleek, abstract superhero avatars, and a darker background color scheme. These features were found to be highly acceptable in formative research conducted in the United States [34,39]. FGD participants nearly unanimously agreed that the theme and design elements were not aligned with app preferences among Thai youth.

I think the color scheme is a little too dark, it should be more colorful and cute...there should be a wider choice of color schemes.

FGD1, Participant 7

The avatars look really mysterious...strange...I want the avatars to look more like us, like ones that can wave...cartoons...that we can choose...or use emojis that look more normal.

FGD1, Participant 5

YMSM also expressed the need for cartoon-style stickers, similar to other mainstream platforms used in Thailand, such as LINE, the most frequently used instant messaging service in Thailand [39]. They noted that they are a prominent part of contemporary communication in Thai culture; hence, they should be featured in a PrEP adherence app.

**Figure 1:**
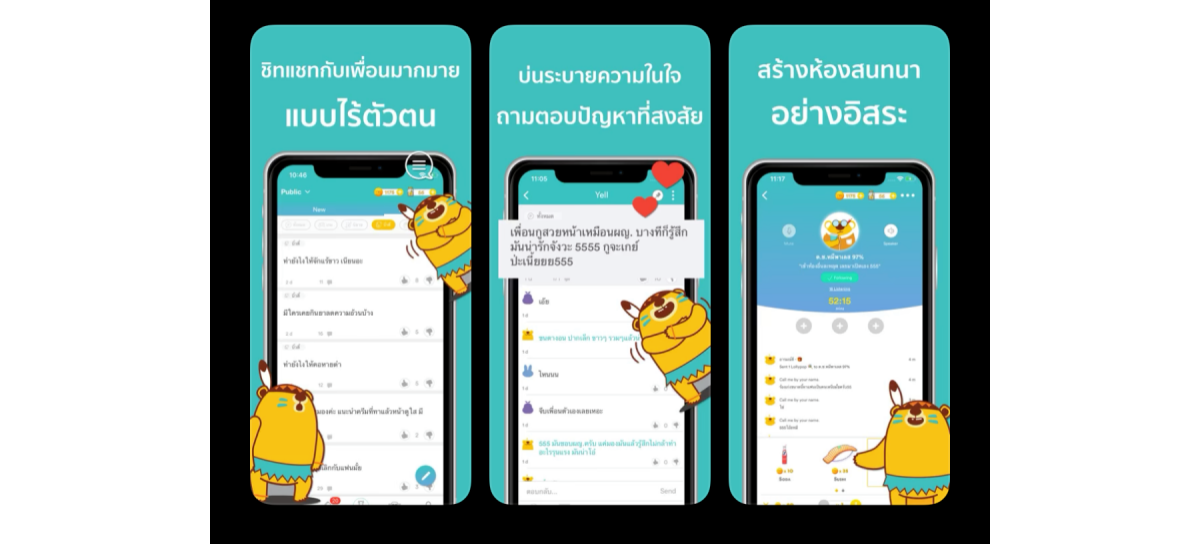
Yellotalk screenshots as displayed in the Apple App Store.

Furthermore, participants indicated a preference for the social wall feature of P3T to have a more attractive look and feel, similar to current popular social networking sites such as YelloTalk (Figure 1):

I want the Social Wall to look like YelloTalk.

FGD 3, Participant 4

##### KII Findings

KII participants commented in a similar way to FGD participants, expressing that youth would likely enjoy a lighter color scheme and a cuter style of graphics:

This looks too depressing and dark...a lighter color theme would be better...the avatars need to be cute cartoons for the kids to pick...

KII, Participant 3

KII participants noted that electronic stickers are a prominent part of contemporary communication in Thai culture; hence, they should be featured in a PrEP adherence app:

Here, everybody is about LINE and stickers. I even engage with senior ministry staff by line stickers. So, it’s not only kids, it’s everybody.

Participant 11

#### Engagement Strategies

##### FGD Findings

FGD participants provided several important recommendations for improving their engagement with the P3T app. First, the app content should be highly dynamic; suggestions include regularly adding new articles, posts about upcoming social events, tailoring to special dates, and current news tailored to individual users and adolescents. Notifications should be sent to alert users to the availability of new content, including contributions from other app users:

If there is an event somewhere it should notify us or notify us about interesting news that will make us want to click in to see. If there is nothing going on in the app and it’s just a boring app, I have several apps on my phone, I would just scroll past.

FGD1, Participant 2

I look at what updates there are, whether they are interesting. There needs to be exchange of knowledge and notifications to make it more interesting, like notifying us a new post has been made on something that interests us, tailoring it to our interests to make us want to use it more.

FGD3, Participant 4

The importance of fun was emphasized by FGD participants, as they highlighted the need for more entertaining features than those found in the reviewed P3 features to optimize engagement:

Something that has a changeable theme, has fun gimmicks, like stress busting games...like the games you can get in Facebook will make the app more appealing.

FGD1, Participant 8

Study participants had varying opinions about rewards that may best incentivize user engagement with the app. Several felt that Thai YMSM would be most interested in earning points for completing app activities that could be redeemed for external rewards such as cash, tickets, or discounts for services.

Apps that I usually use have daily questionnaires...they give point rewards...we get gift vouchers...[we like to exchange points for]...bubble tea, cash, movie tickets, discounts for spas and botox, fastfood, cafes, shoes, buying games.

FGD2, Participant 1

Some FGD participants expressed interest in the choose-your-own-adventure style storytelling collections feature and suggested increasing the adventure and excitement of the storylines. However, to be effective as an incentive for app use, storylines would need to be more exciting and adventurous.

We enjoy reading stories about unique experiences of others...oral sex, group sex...

FGD 1, Participant 2

##### KII Findings

When asked about factors that should be taken into account to maximize engagement in a PrEP adherence app for Thai YMSM, a key informant provided critical insight into the importance of fun in Thai culture:

There is a strong need in the Thai context of interaction for fun. It has to all be about carrots not sticks. About fun. The Thai culture is about—what’s the point if you’re not going to have fun along the way.

KII, Participant 11

A key informant commented specifically about users being limited to redeeming their earned points in P3T to buy choose-your-own-adventure stories in the collections feature. The participant was not optimistic that this approach would incentivize app engagement among Thai YMSM:

Our teenagers don’t have a lot of persistence to do things. If they have to go in to read something, it won’t really motivate them to want to know more, unless it comes in the form of a game, and they get points for doing it...point exchange needs to be for something they want...using points to buy something to read [referring to Collections], I don’t know, that’s not really in the nature of Thai teenagers to do something like that.

KII, Participant 9

#### Privacy Risks

Many FGD participants expressed concerns about sharing their private information on the web and emphasized their preference for personal interactions with health care providers:

I would rather talk to doctors and counselors than tell my personal things to friends that I don’t know.

FGD4, Participant 6

On the basis of the fear of private information being compromised, some FGD participants expressed the need for advanced methods of user authentication:

I want a highly secure system, maybe use a finger or face scan...to access the app...because our medication tracking information and blood results are sensitive information.

FGD1, Participant 4

#### Connectivity Challenges

A main challenge specific to the use of mHealth apps in Thailand is reliable internet access. Many apps, including P3T, require a continuous active internet connection to function. However, internet connectivity is not readily available or affordable for many Thai adolescents. The FGD participants noted the importance of offline app functionality:

For me, like if I don’t have internet this week, or I haven’t bought internet time or haven’t gone home this week, I can fill in my questionnaire responses...and when I’m back online, my survey responses are sent.

FGD 4, Participant 8

#### Recommendations for New App Functions

##### FGD Findings

In FGDs, two additional app functions were discussed most frequently and recommended for inclusion in the P3T. The first recommendation was to add a search function that allowed users to search all of the app’s content:

When I do an internet search [for information] there are so many websites, and sometimes they are focused on selling you things...it takes ages to find the information I actually want...information in an app will get this to information I want directly.

FGD3, Participant 4

The second recommendation was to include a function that allows users to monitor and plan a healthy lifestyle. For example, FGD participants wanted the ability to track HIV risk behaviors in the app and receive customized information about their HIV risk level based on their tracked data:

I want it [the app] to help determine [my risk] based on data I have inputted...if I had a risk event today...how should I deal with it...and use it to go through events with my doctor when I next visit them.

FGD1, Participant 5

##### KII Findings

Key informants pointed out that Thai YMSM appreciate health-related feedback from adolescent-friendly providers, and offering individually tailored information through P3T could be valuable:

One of the things we hear back from some of our participants in research is that they like having health information. We noticed that they’re interested in coming back to their visits so they can get health information and feedback on how they’re doing health wise. I think that can potentially be interesting to people. “Your kidney health is great” and “you don’t have any evidence of STI that’s awesome” or “keep up the good work” or whatever it is.

KII, Participant 11

#### Adaptations Made for P3T Based on Formative Research Findings

On the basis of formative research findings and the limited resources available for adaptations, we prioritized changes to app features while keeping the same content to address the presentation of information provided in P3T and some identified stylistic preferences. These included (1) changing the information presentation style in the knowledge center, (2) adapting stories and images in the collections feature, and (3) changing avatar options. Longer-term structural changes that were not immediately feasible because of resource constraints should be considered in future updates to the app.

#### Presentation Style Changes in the Knowledge Center

**Figure 2:**
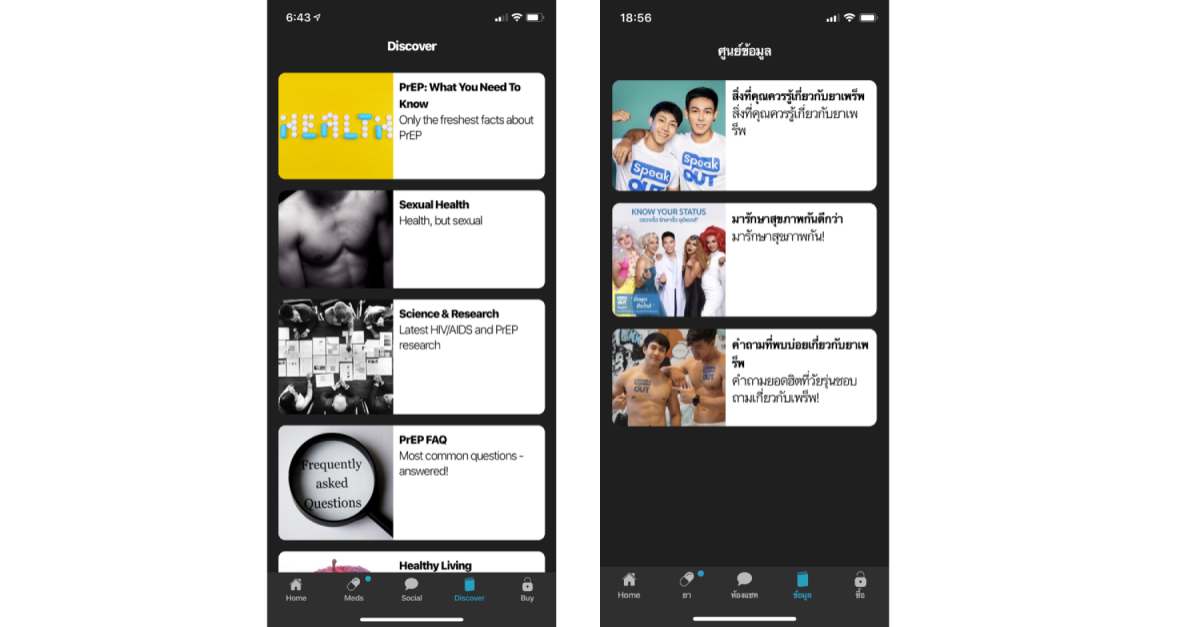
Comparison of knowledge center chapter images before (left) and after (right) adaptation for the Thai context.

**Figure 3:**
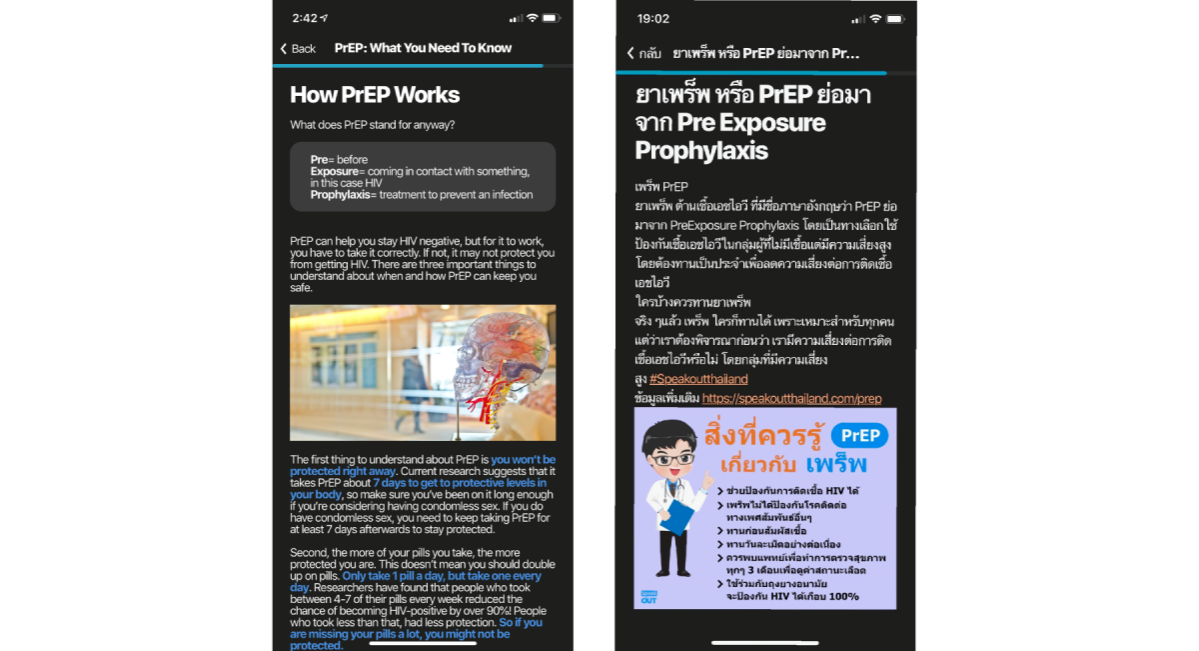
Comparison of knowledge center article on pre-exposure prophylaxis adherence before (left) and after (right) adaptation for the Thai context, including infographics.

**Figure 4:**
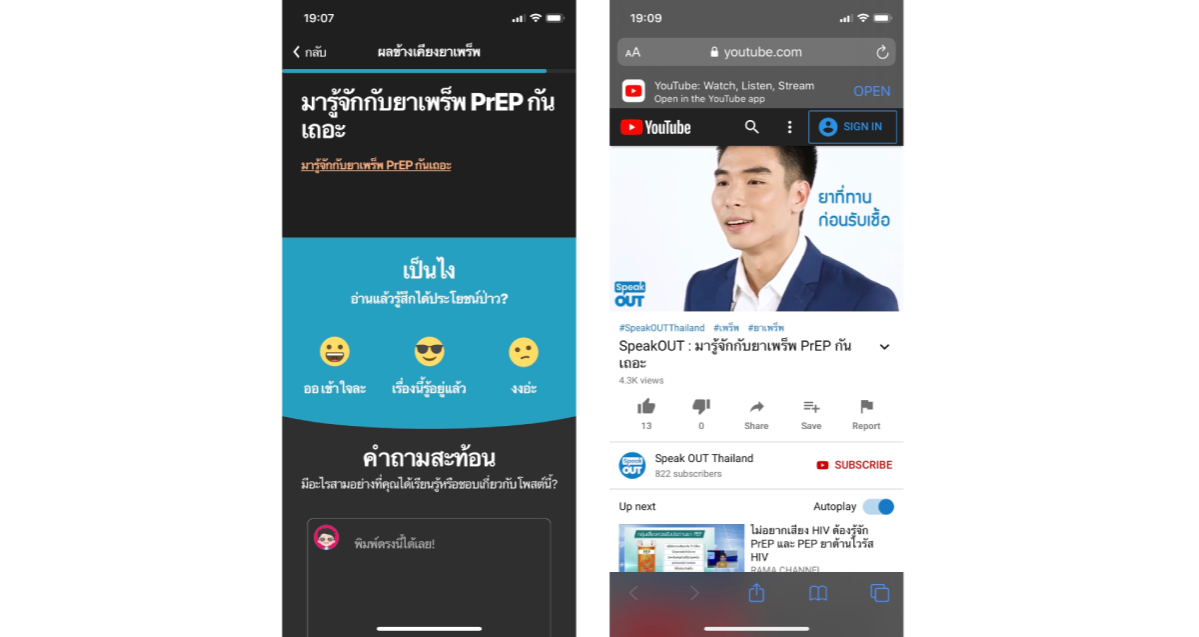
Postadaptation knowledge center page (left) with link to external video content (right).

On the basis of feedback, we changed our information presentation style by creating chapter images more relevant to the Thai context (Figure 2), changing the text-based presentation of information to include less text and more infographics (Figure 3), and added links to outside video content, including those featuring social influencers (Figure 4). All of these videos were locally produced by the Thai HIV prevention NGO, Love Foundation, and reflected popular Thai presentation styles.

#### Adaptation of Stories and Images in the Collections Feature

**Figure 5:**
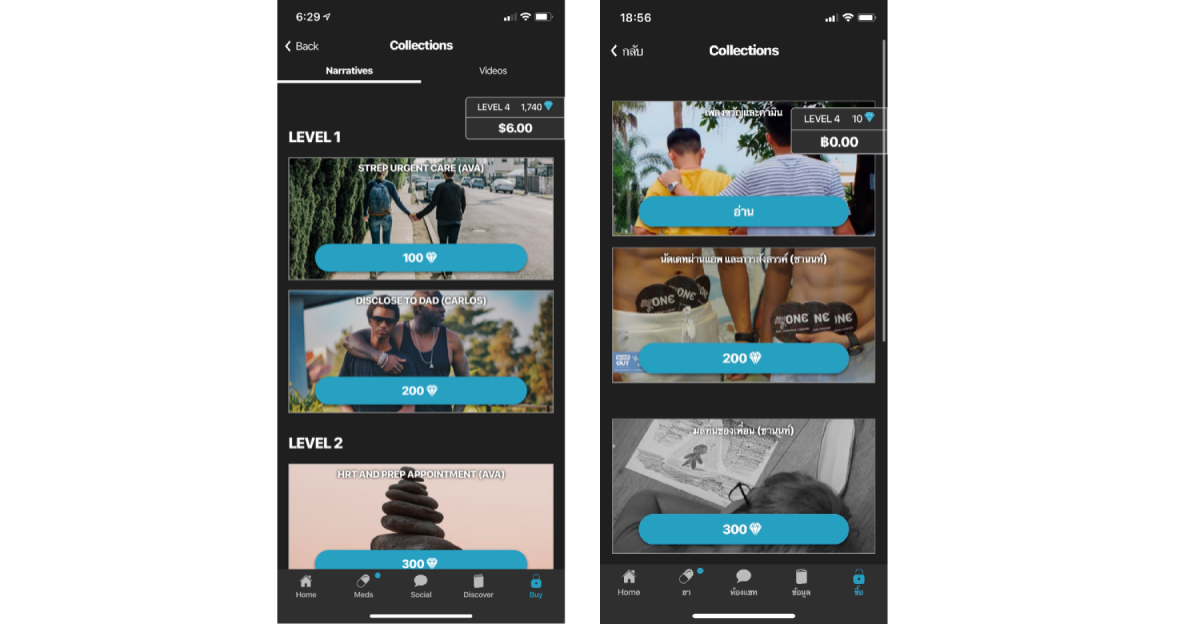
Comparison of Collection titles before (left) and after (right) adaptation for the Thai context.

The collections feature was adapted by giving story characters Thai names and locations and changing some story lines to increase relevance for Thai YMSM. In addition, image icons for the collection titles were changed to Thai characters (Figure 5).

#### Avatar Adaptations

**Figure 6:**
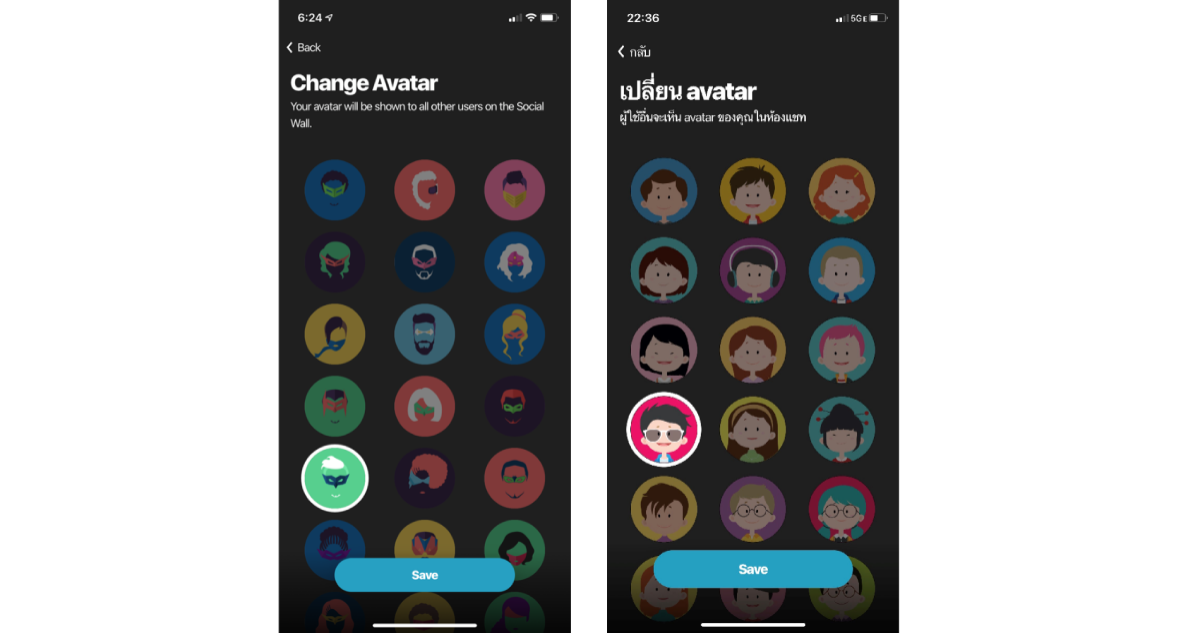
Comparison of avatars before (left) and after (right) adaptation to the Thai context.

As indicated by many FGD participants, the original avatars presented did not appeal to Thai YMSM and were considered a major detraction of the app. We partnered with Thai graphic designers to design a new set of avatars to replace the original avatars. Figure 6 compares the older version and the new version of avatars.

#### Existing P3 Features That Foster PrEP Adherence Facilitators, Address Adherence Barriers, and Align With Key Recommendations

In addition to the specific changes made as indicated in the previous section, many P3 features already address PrEP adherence facilitators and barriers and general app recommendations identified in FGDs and KIIs. For example, P3 seeks to increase PrEP adherence facilitators by providing content that promotes accurate self-assessment of HIV risk via knowledge center content, promoting the exchange of support and information on topics related to PrEP adherence via the social wall, and offer personalized adherence support from a trained adolescent-friendly adherence counselor through the in-app counseling feature.

The knowledge center and social wall also already address some of the general app recommendations related to the reliability of information, access to information, and peer information sharing. The knowledge center is designed to provide reliable information in an easy-to-understand manner. The social wall provides a space for users to discuss topics related to health anonymously. In addition, in terms of security and privacy concerns, the P3 app provides an in-app log-in option for the user to secure the app either by using a personalized PIN number for all platforms or using face recognition technology for iOS platform users when launched. The app helps to protect personal identity by asking users to set their own pseudonyms that do not contain any identifying information about them rather than using their real names in the app. A summary of key study findings and adaptations made is made in Table 1.

**Table 1:** Summary of key study findings and adaptations made.

Findings			Relevant quotes		Adaptations made
**Presentation style preferences**					
	Images more reflective of Thai YMSM^a^ settings Preference for more visually based information and less text Preference for information from social influencers Preference for a cuter style and lighter color theme Content needs to reflect their local cultural context	“I want the avatars to look more like us.” [FGD^b^1, P^c^7] “I think [information] should be [presented as] small nuggets of information...an infoographic, like a poster with concise infographic.” [FGD1, P1] “I think having influencers on there with different messages.” [KII^d^, P11] “A lighter color would be better...the avatars need to be cute cartoons.” [KII, P3] “I want the Social Wall to look like YelloTalk.” [FGD3, P4]		Knowledge center Graphics incorporated more photos taken in the Thai YMSM context More infographics and less text used Links added to outside video content containing messages from social influencers Collections Image icons changed to Thai characters Story characters given Thai names and locations Avatars Created by Thai graphics designer with cute theme and lighter color scheme	
**Facilitators**					
	High HIV risk perception, PrEP cost support, social support	“I used my [risky] history to determine my risk.” [FGD1, P3] “It really helps with us being students [that PrEP is free.” [FGD4, P6] “Some couples who both take PrEP are open to teach other.” [KII, P14]		No adaptations made as same facilitators and barriers as found in P3 US study results	
**Barriers**					
	Stigma, logistical issues, PrEP side effects, low risk perception, and peer influence	“People look at gay men negatively as it is.” [FGD4, P5] “We have a lot of classes...there isn’t always time to come for appointments.” [FGD2, P3] “I had a lot of side effects...makes it impossible to study.” [FGD3, P1] “Some came because they saw their friends taking PrEP...but confess they don’t have time for appointments.” [KII, P2]		No adaptations made to current version because of budgetary restrictions	
**New app function recommendations**					
	Search function and risk behavior tracker	“Information in an app to get information I want directly.” [FGD3, P4] “I want it to help determine [my risk].” [FGD1, P5]		No adaptations made to current version because of budgetary restrictions	

^a^YMSM: young men who have sex with men.

^b^FGD: focus group discussions.

^c^P: participant.

^d^KII: key informant interview.

## Discussion

### Overview

This study explored facilitators and barriers related to PrEP adherence and elicited general and specific recommendations from Thai YMSM and key informants to ensure that adaptations of a PrEP adherence app originally developed for YMSM in the United States were relevant, engaging, and useful for the Thai context. To our knowledge, this is one of the few studies conducted outside high-income settings that address the adaptation of an mHealth approach to support PrEP adherence among YMSM [40].

The individual, societal, and systemic level facilitators and barriers to PrEP adherence among Thai YMSM identified in this study have also been reported in other studies of MSM and YMSM in the United States, Thailand, and other countries. Consistent with other studies [7,41-45], adherence facilitators included awareness of HIV risk and desire to protect themselves, concerns related to sexual partners, support from friends, sexual partners, family members, and adolescent-friendly providers, and PrEP affordability (PrEP is provided free of charge through universal health coverage for key populations in Thailand). Key barriers, also identified in other studies [41-44,46-49], included sexuality, HIV and PrEP stigma, attending regular PrEP medical appointments, forgetting to take PrEP daily or while intoxicated, not having PrEP with them at certain times, running out of PrEP, side effects, and low HIV risk perception. Because the PrEP facilitators and barriers identified in this study had been identified in prior research and were already addressed by the original P3 app, its content was already designed to foster these facilitators and address the barriers and consequently no content was changed; only app feature changes were made in adaptations in this trial.

In general, the main features, such as the social wall, daily quests, and collections of the P3 app were found to be acceptable among the study participants. It is likely that this is in part because of the ways in which the key app features address the factors identified by participants in this study as the most important PrEP adherence facilitators and barriers among YMSM in Thailand. For example, most participants expressed sexuality, PrEP, and HIV stigma as barriers to PrEP adherence and social wall of P3T provides an opportunity for positive social interaction and support related to these issues to take place, which has been shown in previous studies to be associated with reduced stigma [50,51].

However, the study participants provided critical recommendations for P3T app adaptations in the way the core features were presented that address cultural and contextual factors specifically relevant to Thai YMSM, which are expected to substantially enhance app acceptability, relevance, user engagement, and utility. For example, Thai YMSM voiced a strong preference for more visual content, such as infographics and video content, as opposed to text, similar to findings from formative work to develop P3 [34]. This may be because of the interest in consuming information via different mediums [35,36,52-54]. Therefore, these factors highlight the importance of understanding the reading ability and reading norms of the target population when developing app interventions [55]. The desire to keep the app fun and light through the use of stickers, a lighter color scheme, and ongoing seasonal promotions and games also featured prominently in both FGDs and KIIs. These suggestions reflect Thai culture and thus emphasize the importance of understanding cultural preferences and popular marketing practices when adapting mHealth interventions for use in different countries [56-58].

Both YMSM and key informants noted the strong influence peers have on the health beliefs, decision-making processes, and behaviors of Thai YMSM. This phenomenon has been identified in research in other cultures [59,60], and a study of Thai YMSM found that health beliefs and self-efficacy were associated with HIV prevention behaviors [52]—factors known to be influenced by peer groups [61]—were associated with HIV prevention behaviors [52]. App interventions focused on HIV prevention for Thai YMSM should assess whether functions that acknowledge the importance of peer relationships increase the likelihood of achieving the desired behavior. In P3T, peer interactions are possible via the social wall, and positive peer norms are presented in the videos added to the knowledge center that feature peer influencers. Future development of P3T will include the ability to visually represent the popularity of content among users via functions such as number of likes on comments and most popular topics, which could further capitalize on the influence of peers to promote behavior change.

There have been a small number of mobile apps that have tested YMSM at risk for HIV to support PrEP adherence and HIV prevention, mostly based in the United States. The availability of having reliable information on HIV and sexual health, reminder features for users to access relevant health care such as drug adherence and medical appointments, esthetically pleasing features for target users, and ease of use are common findings with what was found in this study from multiple studies previously conducted, including the LYNX and MyChoices apps [62-64]. Similar needs for medication reminders and education on their condition have been found in adolescents with other chronic health conditions, such as sickle cell disease [65]. No striking contrasts in YMSM needs using PrEP adherence apps in other studies compared with findings from those found in our study are apparent so far; however, many studies are underway that may produce differing outcomes to what has been seen so far [62,63,66]. Given the future potential scalability of the use of mobile phone apps in supporting PrEP adherence, understanding differences in feature preferences in different contexts, such as with age, gender identity, urban versus rural dwellers, and specific user profiles are valuable in adjusting mobile health technologies to maximize their benefits to specific populations [67]. Given the time limitations with data collection in adapting mobile technologies to user needs, future studies may benefit from sequential adaptive research trial designs to address this challenge.

### Future Directions

We identified the benefits and challenges of adapting P3 for YMSM in Thailand. First, the remarkable similarities in PrEP adherence facilitators and barriers among YMSM in our study, and in other studies, along with the general acceptability of the key features of P3 among our study participants support the growing trend of adapting evidenced-based apps, such as P3, for different cultures and contexts rather than developing new apps from scratch [31]. However, extensive formative research is critical to ensure that an app is appropriately adapted to meet the unique needs and population preferences in different settings [67,68]. Adapting an existing app with a common behavior change goal can increase the speed of app development, reduce costs, and ultimately expedite scalability [31-33].

However, even minor app changes or the addition of a few new features can require significant time and budgetary resources. Planning for a budget that is sufficient for formative research and app adaptations may ensure that the adapted app is culturally and contextually relevant and may shorten the time between adaptation, pilot study, and, if feasible and acceptable, a larger trial. Owing to the limited time and funding of a pilot grant and working with a commercial app developer based in North America, we were unable to implement all design changes that would better align the app with cultural preferences identified in the FGDs and KIIs. These changes focused on stylistic preferences and the inclusion of additional functions within the existing features. The main changes suggested included making the app color scheme lighter, offering customizable skins, adding stickers to enhance communication in the social wall, adding an app search function, adding engagement statistics to visualize content popularity among peers (eg, number of times a knowledge center article or social wall post has been read), adding health behavior tracking features that would allow for determination of HIV risk level based on data entered into the app, and regular content updates to make the app more dynamic. An additional need identified in using an app such as P3T in Thailand is the ability of the more features of the app to function offline. According to the study participants, most Thai YMSM are unable to afford data plans that would allow for consistent and reliable internet access. To maximize access of users to P3T, more extensive offline functionality is critically important in the Thai context. If justified by the findings from the pilot study, the remaining adaptations, along with additional findings anticipated from the pilot study, are planned for a subsequent development phase that could also inform the adaptation of other apps.

Including local designers and developers on the team may help stretch limited resources and ensure that local expertise leads to the cultural adaptation process [68]. For example, through the support of a Thai collaborator skilled in graphic design, we were able to make important design changes to the app that reflected a rich understanding of the esthetic preferences of Thai YMSM. The local designer rapidly developed avatars that were highly responsive to study participant preferences. We also faced unforeseen formatting challenges translating the content of the app from English to Thai, which substantially increased the amount of time required for this stage. Having a fluent Thai speaker on the development team could help address these and similar barriers in the adaptation process. Local app designers and developers also provide a capacity-building opportunity for local collaborators, which can then reduce cost and time during both adaptation and scale-up phases. Given that, particularly in the context of limited resources such as where this study was performed, it must be acknowledged that there are limited data to support the cost-effectiveness of digital health interventions [69]. Such considerations are important when considering future scalability and sustainability to inform policies on investments in this area of health care interventions [70].

In the COVID-19 era, it must also be noted that with its considerable challenges, there are opportunities to optimize digital approaches as well as psychosocial intervention health care delivery in adolescents. Multiple areas regarding this with relevance to future studies related to the P3T app could be explored, including the impact of the app on preventive behaviors, medication adherence, self-management, and cost-effectiveness [71,72].

### Strengths

A major strength of this study was that it addressed a knowledge gap in the feasibility and acceptability of mHealth in low-middle income settings [29] and also in adolescent populations [28]. In addition, it provided practical guidance for adaptation of an existing mHealth platform in low-middle income settings rather than starting from scratch, a practical solution in the context of limited financial and staff resources as well as difficult-to-reach populations. It also highlighted the importance of using local expertise and capacity building in producing well-suited and sustainable interventions where mHealth interventions are adapted to very different settings for which they were originally intended. Finally, this study involved target user input from the start of its adaptation to the Thai context, an approach known to improve target user short- and long-term engagement [65,73-75].

### Limitations

This study was conducted only in Bangkok; therefore, data collected from study participants may not reflect the perspectives of YMSM and PrEP providers nationwide. In addition, the majority of FGD participants were enrolled in full-time education, which in the context of Thailand signifies a higher socioeconomic status. Therefore, these findings may not be generalizable to the Thai YMSM of all socioeconomic groups. As parental consent was required for FGD participants under the age of 18 years, we were unable to enroll participants in this age group because of the unwillingness of potential participants to reveal their sexual orientation or risk behaviors to their parents. This may have led to findings biased toward the views, needs, and developmental requirements of older adolescents. In most countries, formidable challenges remain in conducting research with those under the age of 18 years, despite the need to gather information to inform interventions that address the current global HIV epidemic, which disproportionately affects young people [5,76]. In Thailand, parental consent is exempted for individuals under 18 years of age to receive sexually transmitted infection care, HIV testing, or PrEP initiation. However, the decision to allow those under 18 years to participate in HIV prevention research without parental consent is at the discretion of the ethical review boards. On the basis of our experience, some review boards in Thailand are willing to allow exemption of parental consent for adolescents in HIV prevention trials where study involvement is a direct benefit to participants from this key population.

Despite these limitations, the adapted P3T app represents a significant step toward expansion and scale up of an app-based intervention to support PrEP adherence in Thailand, where PrEP has recently become available free of charge for YMSM through a national health coverage [5,77]. Additional studies in different geographical areas of Thailand with a high YMSM population density and studies focusing on young transgender women who have sex with men and other key populations would help further the adaptation processes of mHealth apps for those disproportionately affected by HIV in Thailand.

### Conclusions

As observed in this study, most facilitators and barriers for PrEP adherence in the Thai context are similar to those observed in other global settings. The core features of the P3T app address the main facilitators and barriers related to PrEP adherence in Thailand for YMSM. The majority of the improvements suggested in this study focus on customizing the app to suit the stylistic presentation preferences of Thai YMSM and tailoring the app content to suit the cultural context of Thailand and the cultural nuances of the YMSM demography. Given that many of the facilitators and barriers are similar globally, adapting existing successful mHealth solutions will be the most effective and cost-efficient way forward as opposed to developing new apps. However, to obtain optimal results, it is vital that local app developers are included as partners in the adaptation process.

## Abbreviations

FGD: focus group discussion
KII: key informant interview
mHealth: mobile health
MSM: men who have sex with men
P3: Prepared, Protected, emPowered
P3T: P3 Thailand
PrEP: pre-exposure prophylaxis
TRCARC: Thai Red Cross AIDS Research Center
VCT: voluntary counseling and testing
YMSM: young men who have sex with men
